# Covalently Functionalized Halloysite-Calixarene Nanotubes for Injectable Hydrogels: A Multicavity Platform for Hydrophobic Drug Delivery

**DOI:** 10.3390/ph18091356

**Published:** 2025-09-11

**Authors:** Giuseppe Cinà, Marina Massaro, Andrea Pappalardo, Carmela Bonaccorso, Cosimo G. Fortuna, Placido G. Mineo, Angelo Nicosia, Paola Poma, Rita Sánchez-Espejo, Caterina Testa, César Viseras, Serena Riela

**Affiliations:** 1 Dipartimento di Scienze e Tecnologie Biologiche, Chimiche e Farmaceutiche (STEBICEF), Università di Palermo, Viale delle Scienze, Parco d’Orleans II, Ed. 17, 90128 Palermo, Italy; giuseppe.cina05@unipa.it (G.C.); marina.massaro@unipa.it (M.M.);; 2 Dipartimento di Scienze Chimiche, Università degli Studi di Catania, 95125 Catania, Italy; carmela.bonaccorso@unict.it (C.B.); cgfortu@unict.it (C.G.F.); placido.mineo@unict.it (P.G.M.); angelo.nicosia@unict.it (A.N.); caterina.testa@phd.unict.it (C.T.); 3 Consorzio Interuniversitario Nazionale per la Scienza e Tecnologia dei Materiali (INSTM), Unità di Ricerca di Catania, 95125 Catania, Italy; 4 Istituto per i Processi Chimico-Fisici, Consiglio Nazionale delle Ricerche (IPCF-CNR), Viale F. Stagno d’Alcontres 37, 98158 Messina, Italy; 5 Department of Pharmacy and Pharmaceutical Technology, Faculty of Pharmacy, Campus Universitario de Cartuja, University of Granada, 18071 Granada, Spain; ritamsanchez@ugr.es (R.S.-E.); cviseras@ugr.es (C.V.); 6 Andalusian Institute of Earth Sciences, CSIC-UGR, 18100 Armilla, Spain

**Keywords:** halloysite, calix[5]arene, laponite hydrogels, drug delivery

## Abstract

**Background**: Poor water solubility is a major limitation for the therapeutic use of many anticancer drugs. In this study, we report the design and development of two halloysite-based hybrid nanomaterials for the encapsulation and delivery of hydrophobic and positively charged drugs. **Methods**: A novel multicavity platform was obtained by covalently grafting calix[5]arene macrocycles onto the external surface of halloysite nanotubes (HNTs), combining lumen encapsulation with supramolecular host–guest recognition. PB4, a planar and hydrophobic pyridinium salt with significant antiproliferative activity, was selected as a model compound. Both PB4-loaded HNTs (HNTs/PB4) and calixarene-functionalized HNTs (HNTs-Calix/PB4) were incorporated into Laponite^®^-based thixotropic hydrogels to obtain injectable and biocompatible systems. **Results**: The nanomaterials were thoroughly characterized, and their loading efficiency, release behavior, and aqueous dispersibility were evaluated. Antiproliferative tests on MCF-7 cells demonstrated that both hydrogels retained PB4 activity, with distinct release profiles: the pristine HNTs allowed faster drug availability, while calix[5]arene-functionalized systems promoted sustained release. **Conclusions**: This work introduces the first example of covalently calixarene-functionalized halloysite and presents a versatile drug delivery platform adaptable to different therapeutic contexts and combination strategies.

## 1. Introduction

Nanotechnology, and thus nanomedicine, are important tools for the development of innovative biomedical applications [1,2,3]. In recent years, the development of nanostructured materials for this purpose has shifted toward systems that are not only efficient in drug loading and release, but also sustainable, biocompatible, and responsive to physiological conditions [4,5]. Organic nanocarriers include liposomes, micelles, dendrimers, polymer conjugates, and cyclodextrin, while inorganic materials comprise iron oxide, gold nanoparticles, and mesoporous silica nanoparticles and are the most employed in nanomedicine [6,7,8,9]. Among natural materials, clay minerals [10], particularly halloysite nanotubes (HNTs), stand out due to their abundance, low cost, and intrinsic biocompatibility [11,12].

Naturally uptaken by cells via endocytosis [13], HNTs have been used as carrier systems for several hydrophobic species [14,15,16,17].

Halloysite is an aluminosilicate with a hollow tubular morphology [18,19,20]. Its dimensions vary based on the geological source, typically ranging from 500 to 1500 nm in length and 10 to 30 nm in diameter, with a general formula of Al_2_Si_2_O_5_(OH)_4_·nH_2_O [21]. Its inner (positively charged) and outer (negatively charged) surfaces allow for a wide range of interactions, while its central lumen provides a confined space for guest encapsulation [22,23]. Used for centuries in traditional Chinese medicine [15,24], HNTs have gained attention as a drug carrier platform, particularly for chemotherapeutic agents [25,26], due to their ability to enhance bioavailability, cellular uptake [27], and antiproliferative activity [28,29].

Despite these advantages, the delivery of highly hydrophobic molecules remains a challenge. These molecules exhibit poor water solubility and often require organic solvents for administration, limiting their biomedical applicability. Although HNTs can host such molecules through lumen encapsulation, their aqueous dispersibility often results in aggregation and diminished therapeutic performance [24,30]. To overcome this limitation, recent research has focused on the surface functionalization of HNTs with macrocyclic receptors. In particular, multicavity systems combining the inner lumen of HNTs with external host units such as cyclodextrins have shown enhanced loading efficiency, stabilization of guest molecules, and better performance in aqueous media [31,32]. Beyond cyclodextrins, other macrocyclic hosts such as calixarenes [33,34,35], pillararenes [36], and so on have also emerged as promising candidates interacting with highly hydrophobic molecules through hydrophobic interactions and π–π stacking [37]. In particular, calix[5]arenes have demonstrated the ability to form multivalent and directional binding architectures, making them especially suitable to the encapsulation of various guest molecules. Their strong binding affinity for quaternary ammonium species and other cationic aromatic compounds arises from a combination of electrostatic attraction and π–π interactions. This makes them highly attractive for the development of nanocarriers targeting polar, hydrophobic drugs [38,39].

However, despite significant progress, currently available hybrid nanomaterials still face major limitations in the targeted delivery of hydrophobic and positively charged drugs. These include low encapsulation efficiency, poor control over release kinetics under physiological conditions, limited colloidal stability, and a tendency to aggregate in aqueous environments [24,30]. Furthermore, delivery strategies based solely on physical adsorption often fail to ensure drug retention, leading to premature release and reduced therapeutic efficacy [40]. Specifically, lipid–polymer hybrid nanoparticles (LPHNPs), though promising for hydrophobic drug delivery, still exhibit limitations in stability and release control [41]. Additional reviews on nanocarrier limitations and design challenges further emphasize the necessity for multifunctional platforms [42,43]. These observations underscore the urgent need to develop multifunctional nanocarriers that integrate complementary binding and release mechanisms to enhance stability, delivery efficiency, and therapeutic control.

To address the challenge of poor aqueous dispersibility of hydrophobic drugs, we developed a novel multicavity hybrid system based on halloysite nanotubes covalently functionalized with calix[5]arene units. This work represents, to the best of our knowledge, the first report of covalent modification of halloysite with calixarene macrocycles, establishing a new class of hybrid materials designed to enhance the encapsulation, dispersion, and controlled release of highly hydrophobic, planar compounds.

As a proof-of-concept, we selected *E*,*E*-[2,6-di-(*p*-dimethylamino)styryl]-1-methylpyridinium salt (PB4) [44], a planar aromatic compound featuring a permanent positive charge on the pyridinium ring and a highly hydrophobic character, structural features that make it an excellent candidate for calixarene-based host–guest systems. In addition, PB4 has demonstrated antiproliferative activity against MCF-7 breast cancer cells, A549 and H226 lung tumor cell lines, and CaCo2 colon–rectal adenocarcinoma cells [45], outperforming 5-fluorouracil in vitro [44]. However, its poor water solubility limits any in vivo application. To evaluate the impact of calixarene functionalization, we compared two systems: PB4 loaded into pristine HNTs (HNTs/PB4), and PB4 loaded into calix[5]arene-functionalized HNTs (HNTs-Calix/PB4). This comparative approach allowed us to assess the synergistic role of external calixarene cavities in improving PB4 encapsulation, aqueous stability, and release behavior under physiological conditions

The functionalization of HNTs with calix[5]arene units was achieved via Cu(I)-catalyzed azide–alkyne cycloaddition (“click” chemistry), yielding a multicavity nanocarrier in which the halloysite lumen and calixarene cavities synergistically enhance molecular recognition and aqueous stability. As a model system, we selected PB4 for its well-known biological activity and poor water solubility. The resulting nanomaterial was characterized in terms of morphology, loading capacity, colloidal behavior, and release profile under physiologically relevant conditions.

Successful PB4 loading was verified by thermogravimetric analysis (TGA) and FT-IR spectroscopy, the halloysite interacting surface was established by adsorption isotherm, and the morphology of the HNTs/PB4 nanomaterial was imaged by high-angle annular dark field scanning transmission electron microscopy (HAADF/STEM) coupled with energy-dispersive X-ray spectroscopy (EDS). The aqueous mobility was monitored by dynamic light scattering measurements, and the surface charge was evaluated by ζ-potential analysis. To translate these nanomaterials into injectable formulations, both pristine and calixarene-modified HNTs were embedded in Laponite^®^-based thixotropic hydrogels [46], which offer shear-thinning, self-healing properties, and controlled release [47,48,49].

In vitro cell viability assay confirmed the therapeutic potential of the resulting hybrid hydrogels as delivery platforms for poorly soluble anticancer agents. Overall, this study introduces a new class of halloysite-based nanomaterials that combines supramolecular recognition, aqueous dispersibility, and injectability, paving the way for biomedical applications of otherwise intractable hydrophobic drugs like PB4.

## 2. Results and Discussion

In order to develop a supramolecular nanocarrier system to efficiently deliver the hydrophobic PB4 molecule for potential biomedical applications, two different nanomaterials were developed. The first is based on the supramolecular loading of PB4 onto pristine HNTs via hydrophobic effects or electrostatic interactions, resulting in the HNTs/PB4 nanomaterial. The second is based on PB4 loading onto a multicavity halloysite-based system obtained by the covalent linkage of propargyl calix[5]arene (5-Propargyl-11,17,23,29-tetrakis(1,1-dimethylethyl)-31,32,33,34,35-penta(4-methylpentyloxy)calix[5]arene) onto azido-modified HNTs, affording the HNTs-Calix/PB4 nanomaterial.

### 2.1. Synthesis of HNTs/PB4 Nanomaterial

**Scheme 1:**
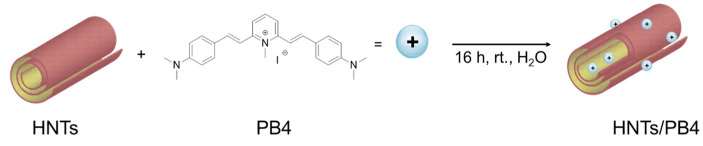
Schematic representation of synthesis of HNTs/PB4 nanomaterial.

The loading of PB4 into halloysite was carried out by a procedure reported elsewhere [32]. In detail, a dispersion of HNTs in water was mixed with a saturated solution of PB4 in ACN. In these conditions, because of the very low aqueous solubility of PB4, it was hypothesized that it could interact both with the inner surface of the HNTs by hydrophobic effects and with external one by electrostatic interactions (Scheme 1). After the work-up, the amount of PB4 loaded onto HNTs/PB4 nanomaterial, estimated by TGA, was ca. 1.4 wt% with an entrapment efficiency of 28%.

**Figure 1:**
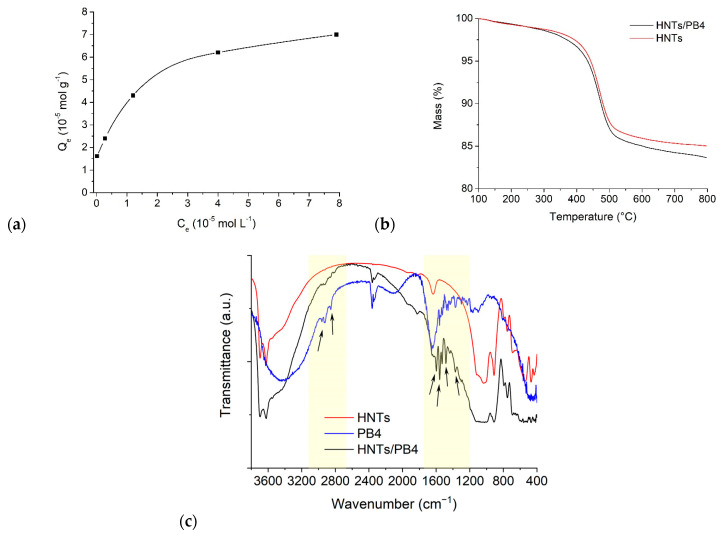
(**a**) Adsorption isotherm of PB4 on HNTs in water at 25 °C; (**b**) thermogravimetric curves of HNTs and HNTs/PB4 nanomaterial; (**c**) FT-IR spectra of HNTs, PB4, and HNTs/PB4 nanomaterials.

To confirm which surface of the HNTs interacts with the organic molecule, an adsorption isotherm experiment was performed (Figure 1a). In particular, the equilibrium amount of PB4 adsorbed into the clay (Q_e_, mol g^−1^) as a function of the equilibrium PB4 concentration in solution (C_e_, mol L^−1^) is reported. As it is possible to observe, the amount of adsorbed molecules increases by increasing the equilibrium PB4 concentration. To better understand the interaction mode of PB4 onto HNTs, the experimental data were analyzed by Langmuir and Freundlich models. The results obtained are reported in Table 1. As it is possible to observe, they were better fitted by the Freundlich model, indicating thus heterogeneous and multimolecular layer adsorption [50], in agreement with the interaction of the heteroaromatic stilbene derivative with both halloysite surfaces. Since the found *n* value is greater than 1, the adsorption is favorable, and it could be ascribed to a physical process [51].

The HNTs/PB4 nanomaterial was characterized by FT-IR spectroscopy and TGA, and the colloidal properties were estimated by DLS and ζ-potential measurements. In addition, its morphology was investigated by transmission electron microscopy (TEM) coupled with energy-dispersive X-ray spectroscopy (EDX).

Figure 1b reports the TG curve of the HNTs/PB4 nanomaterial and, for comparison, that of pristine HNTs, normalized for the water content. The TG curve of the HNTs/PB4 nanomaterial shows the typical mass loss of halloysite due to the removal of interstitial water molecules, between ~300 and 500 °C [52,53]. In the same range of temperature, the decomposition of the PB4 likely occurs. From the analysis of the residual matter at 800 °C, it was possible to calculate the percentage loading of PB4 onto the HNTs discussed above.

FT-IR spectroscopy further confirms the presence of PB4 in the HNTs/PB4 nanomaterial. In Figure 1c, the FT-IR spectrum of the HNTs/PB4 nanomaterial and those of pristine components HNTs and PB4 are reported. Vibration bands related to pristine halloysite are assigned based on the literature data [54]. Besides these, other vibration bands are also clearly observable in the nanomaterial; these are related to the stretching and bending of aromatic and aliphatic tertiary amines in the range between 1370 and 1200 cm^−1^ and some signals between 1620 and 1500 cm^−1^, which correspond to the stretching vibration of the C=C bond of aromatic rings attributable to the PB4 molecules.

By measuring the average translational diffusion coefficient (D) and tracking the mobility of the nanomaterials in water, DLS measurements enable the determination of the structural properties of the nanomaterials. This coefficient considers the diffusing particles’ dimensions, shapes, and levels of hydration, in addition to the presence of aggregation phenomena. The HNTs/PB4 nanomaterial showed a D value of 4.9 × 10^−13^ m^2^ s^−1^, lower than that of pristine HNTs (1.6 × 10^−12^ m^2^ s^−1^), indicating a slow diffusion in aqueous media as a consequence of the introduction of hydrophobic species onto the external surface of the HNTs. This evidence was further confirmed by a polydispersity index of 1.0, indicating a broad size distribution of diffusing objects consistent with the presence of some aggregation phenomena in aqueous environment [55].

ζ-potential measurements agree with DLS data showing a ζ-potential value for HNTs/PB4 nanomaterial of ca. −5.5 ± 1.0 mV, more positive than that of pristine HNTs (−18.0 ± 1.0 mV) as a consequence of the partial neutralization of the negatively charged external surface of the HNTs after interacting with PB4.

**Figure 2:**
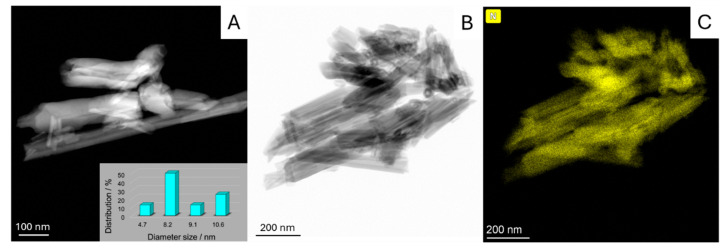
(**A**,**B**) HAADF/STEM image of HNTs/PB4 nanomaterial; (**C**) HAADF/STEM of HNTs/PB4 nanomaterial with elemental mapping images. Inset shows HNTs/PB4 diameter size distribution (*n* = 20).

The morphology of the HNTs/PB4 nanomaterial was imaged by high-angle annular dark field scanning transmission electron microscopy (HAADF-STEM). The HAADF/STEM image (Figure 2A,B) of the nanomaterial showed that the structure of the HNTs was preserved after loading of PB4. The tubes seem to be aggregated according to the presence of organic molecules onto the external surface of the HNTs, as shown by the elemental mapping, highlighting N atoms, extrapolated by energy-dispersive X-ray spectroscopy (EDS) (Figure 2C). On the other side, loading PB4 into the lumen HNTs is shown. Indeed, the lumen is not apparent in all its length and exhibits a decreased diameter, i.e., from 14.9 nm for pristine HNTs (Figure S2) to ca. 8.5 ± 1.5 nm (Figure 2A).

### 2.2. Synthesis of HNTs-Calix/PB4 Nanomaterial

**Figure 3:**
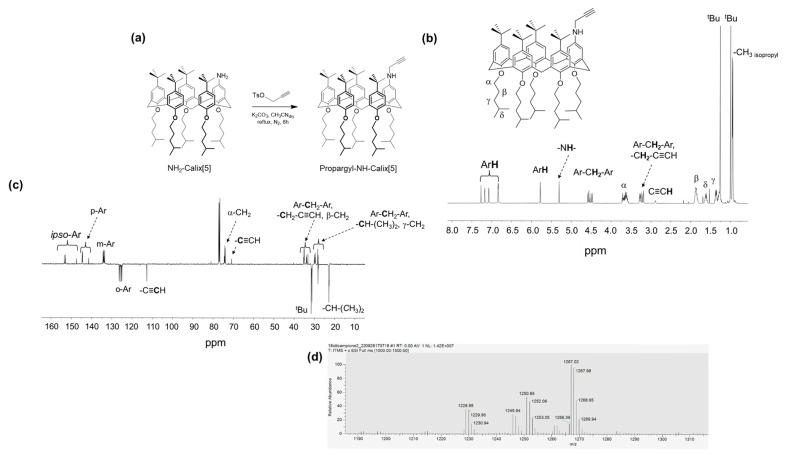
(**a**) Schematic representation of synthesis of Propargyl-NH-Calix[5]; (**b**,**c**) ^1^H and ^13^C NMR spectra of Propargyl-NH-Calix[5] recorded in CDCl_3_; (**d**) ESI-MS spectrum of Propargyl-NH-Calix[5] in CH_3_OH/H_2_O (4:1, *v*/*v*).

The synthesis of propargyl calix[5]arene was achieved starting from NH_2_-Calix[5]. This was synthetized through a series of synthetic steps starting from *p-tert*-butylphenol and formaldehyde (see Supplementary Information) [39,56]. The transformation of NH_2_-Calix[5] into the corresponding propargylated derivative was achieved via *N*-alkylation using propargyl tosylate under basic conditions. The reaction was carried out in the presence of a mild base (e.g., K_2_CO_3_) in a polar aprotic solvent such as acetonitrile, allowing for the selective substitution of the amino group without affecting the rest of the macrocyclic scaffold (Figure 3a). The success of the reaction was confirmed by the appearance of characteristic signals in the ^1^H and ^13^C-NMR and MS spectra (Figure 3b, Figure 3c, and Figure 3d, respectively). The final product, Propargyl-NH-Calix[5], was isolated as a yellowish powder (48% yield) and represents a novel functionalized calixarene bearing terminal alkyne groups suitable for future click chemistry.

**Scheme 2:**
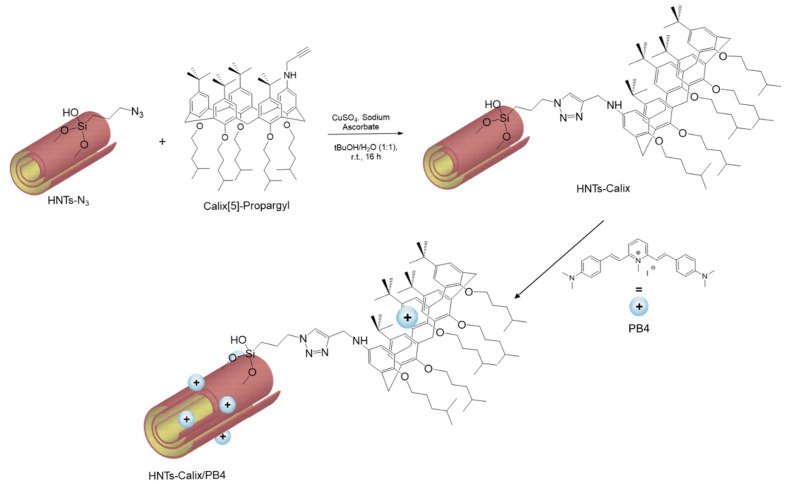
Schematic representation of synthesis of HNTs-Calix/PB4 nanomaterial.

HNTs-Calix material synthesis was achieved by the Meldal–Sharpless–Huisgen azide-alkyne 1,3 dipolar cycloaddition between azido-modified halloysite (HNTs-N_3_) and Propargyl-NH-Calix[5], bearing terminal alkyne groups (Scheme 2). The covalent grafting of calix units onto HNTs-N_3_ was performed at 100 °C for 10 min, in the presence of CuSO_4_ and sodium ascorbate as catalysts, in a mixture of H_2_O/^t^BuOH (1:1) as the solvent, under microwave irradiation. After work up, the loading, estimated by TGA, was as large as of 4.1 wt%. Subsequently, the HNTs-Calix nanomaterial was loaded with PB4 molecules following the approach described above. The loading of the molecule onto the carrier was estimated as 4.7% with an entrapment efficiency of 96%, similarly to that already reported in the literature [32].

**Figure 4:**
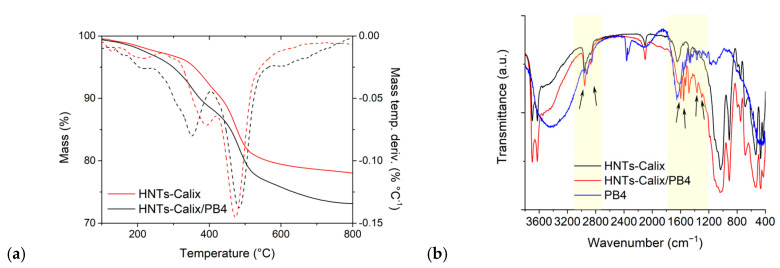
(**a**) TGA (continuous lines) and DTG (dashed lines) curves and (**b**) FT-IR spectra of HNTs-Calix and HNTs-Calix/PB4 nanomaterials.

Figure 4a shows the thermogravimetric curves of HNTs-Calix and HNTs-Calix/PB4 nanomaterials. As one can see, besides the typical mass losses of halloysite arising from the expulsion of the interlayer water molecules (centered ca. 550 °C), in the HNTs-Calix/PB4 differential thermogravimetric curve, a broad peak centered at ca. 350 °C is present, which is different from that related to degradation of calixarene in the HNTs-Calix nanomaterial, providing further evidence of the PB4 loading onto the carrier [53]. In Figure 4b, the FT-IR spectrum of the HNTs-Calix/PB4 nanomaterial is reported. By comparing it with the one of HNTs-Calix, it is possible to observe the presence of the typical vibration bands of PB4, confirming the successful loading.

DLS and ζ-potential measurements showed that the HNTs-Calix/PB4 nanomaterial possesses a Z-average size greater than that of HNTs-Calix (>2000 nm and >1000 nm for HNTs-Calix/PB4 and HNTs-Calix, respectively), indicating that PB4 interacts both with the calix[5]arene cavity and HNT surfaces, particularly the external one as observed in the case of the HNTs/PB4 nanomaterial. These findings were also confirmed by the obtained ζ-potential value of ca. −9.0 mV, more positive than the one of HNTs-Calix (−15.0 mV).

### 2.3. Photophysical Properties of HNTs/PB4 and HNTs-Calix/PB4 Nanomaterials

It is well-known that stylbazole salts with dimethylamine substitutes possesses interesting photophysical properties arising from their push–pull character due to the presence of a strong electron acceptor in their skeleton, the pyridinium group, and a strong donor, the dimethylanilino group [57,58].

**Figure 5:**
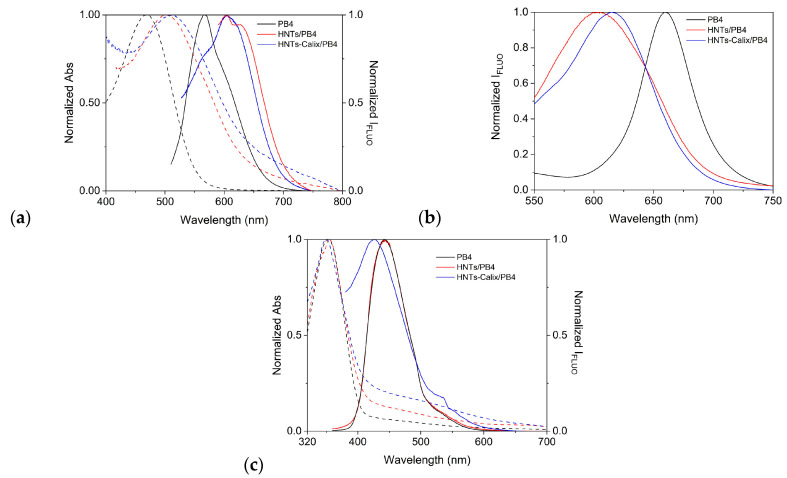
(**a**) UV-*vis* (dotted line) and emission (continuous line) spectra of PB4 (30 μM), HNTs/PB4 (1 mg mL^−1^) and HNTs-Calix/PB4 (1 mg mL^−1^) nanomaterials in water (pH 7.0) at 25 °C; (**b**) solid state fluorescence spectra of PB4, HNTs/PB4, and HNTs-Calix/PB4 nanomaterials; (**c**) UV-*vis* (dotted line) and emission (continuous line) spectra of PB4 (30 μM), HNTs/PB4 (1 mg mL^−1^), and HNTs-Calix/PB4 (1 mg mL^−1^) nanomaterials in HCl 1.0 N at 25 °C.

The UV-*vis* spectra of aqueous HNTs/PB4 and HNTs-Calix/PB4 dispersions (1 mg mL^−1^) show an intense absorption maximum at ca. 500 nm (Figure 5a); in comparison to pristine PB4 (λ_max_ = 470 nm), the maximum absorbance values of the nanomaterials are red shifted of ca. 30 nm, likely due to the confinement of the organic molecule on HNT-based nanomaterials, which provide a more apolar system than the sole water [59].

In line with these findings, by illuminating both dispersions at 500 nm, an intense emission at ca. 630 nm is observed, conversely to the pristine molecule, which shows an emission centered at ca. 560 nm. Solid state fluorescence spectra of the HNTs/PB4 and HNTs-Calix/PB4 nanomaterials (Figure 5b) showed an emission band at ca. 600 nm, while pristine molecules show emission at ca. 660 nm. This behavior indicates that the emission of both HNTs/PB4 and HNTs-Calix/PB4 is not affected by the absence of solvent.

In water, the luminescence quantum yield of PB4, calculated by using fluorescein as the standard, is about 3%, while it increases to ca. 8 and 20% in the case of PB4 in HNTs/PB4 and HNTs-Calix/PB4 nanomaterials, respectively. These findings further confirm that the development of halloysite-based carriers, especially multicavity ones, such as the HNTs-Calix nanomaterial, is a good strategy to improve the physico-chemical properties of the molecule.

By changing the pH of the medium, thus recording the UV-*vis* spectrum of the HNTs/PB4 and HNTs-Calix/PB4 dispersions in HCl (1 N), we observed a bathochromic shift in the absorption band of ca. 350 nm with an emission at ca. 490 nm. These spectra are similar to that of pure PB4, indicating that in acidic media, the influence of the carrier on spectroscopic properties is negligible (Figure 5c). The blue shift observed in comparison to neutral pH is ascribed to protonation of the dimethylamine group, causing the push–pull character of the monocationic PB4 to be lost [60].

### 2.4. Kinetic Release Experiments

**Figure 6:**
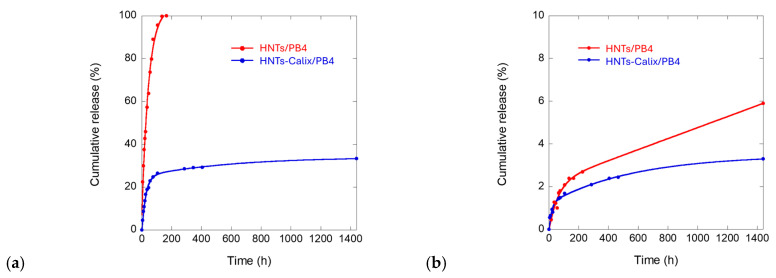
Kinetic release of PB4 from HNTs/PB4 and HNTs-Calix/PB4 nanomaterials in (**a**) HCl 0.1 M, pH 1.0; (**b**) water at pH 7.0 and 37 °C.

To evaluate the performances of the obtained nanomaterial for application in the biomedical field, the kinetic release of PB4 molecules from HNTs/PB4 and HNTs-Calix/PB4 nanomaterials was evaluated by the dialysis bag method at pH 1.0, to understand the behavior of the nanomaterial in acidic media, and at 7.0 and 37 °C, to mimic physiological conditions. The obtained kinetic data are reported in Figure 6. As it is possible to observe, the release of PB4 from the HNTs/PB4 nanomaterial was very fast, reaching the 100 wt% of molecules released after ca. 200 min at pH 1.0 (Figure 6a, red line), whereas a very small amount of PB4 was released from the HNTs/PB4 nanomaterial under neutral conditions (Figure 6b, red line) within 24 h. The fast release at acidic pH could be explained as follows: at pH 1.0, PB4 is fully protonated (pK_a_ = 2.48) [60], whereas HNT is below its isoelectric point (pH ca. 6.5) and in such acidic medium, it is almost fully protonated [61], whereby electrostatic repulsions with the positively charged PB4 could exist, accelerating the release.

Conversely, the kinetic release of PB4 from HNTs-Calix/PB4 showed a slow release at pH 1.0 (Figure 6a, blue line), where ca. 30 wt% of the total amount of loaded PB4 is released from the carrier at pH 1.0 within 24 h. The slower release of PB4 observed in the case of HNTs-Calix/PB4 in comparison to that from HNTs/PB4 could be due to the affinity that PB4 possesses for the calix[5]arene cavity [38,39]. Under neutral conditions, both nanomaterials showed a very slow release of PB4 from the carriers, where ca. 5 wt% of the total amount of loaded molecules was released in 24 h, in agreement with the very low solubility of PB4 in aqueous medium (Figure 6b, red and blue lines).

The kinetic data were analyzed by different models, namely first-order, Power Fit, and the double exponential model (DEM) to obtain information about the release mode. The obtained kinetic data, reported in Table 2, showed that the release of PB4 from HNTs/PB4 nanomaterial, at both pH, follows the first-order model, indicating a diffusion of the molecule from the carrier. On the contrary, the kinetic release of PB4 from the HNTs-Calix/PB4 nanomaterial is better described by DEM, indicating diffusion from different interacting surfaces, thus from both HNT surfaces and calix[5]arene cavity, at both pH investigated. Indeed, the two kinetic constants found are in agreement with the above hypothesis, particularly since *k*_2_ < *k*_1_, it is possible to conclude that we expected a very slow release from the calix[5]arene cavity (*k*_2_) due to the strong interaction that occurs between PB4 and the macromolecular host, followed by a faster release from the surfaces of the HNTs. Similar behavior was observed in the literature for the different release modes of an anionic and non-ionic drug from carbon nanotube surfaces [62].

### 2.5. Development of Inorganic Hydrogels for Potential Biomedical Applications

**Figure 7:**
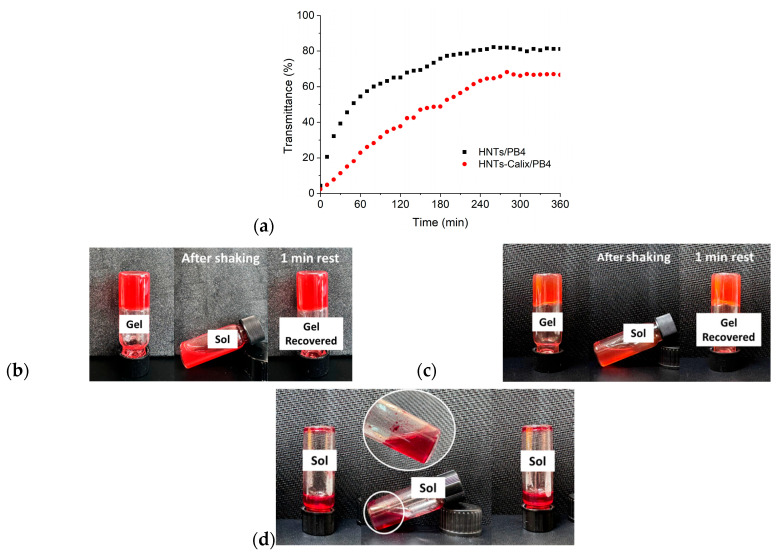
(**a**) Transmittance as function of time for HNTs, HNTs/PB4, and HNTs-Calix/PB4 dispersions in water. Nanomaterial concentration is 1 mg mL^−1^ in all cases; images of obtained hydrogels in presence of (**b**) HNTs/PB4; (**c**) HNTs-Calix/PB4, and (**d**) PB4 as fillers.

As is known, PB4 is sparsely soluble in water and its presence on the external surface of the HNTs in both developed nanomaterials could hamper their actual utilization in the biomedical field. As discussed above, both nanomaterials showed a low diffusion in aqueous media (as highlighted by DLS measurements). Furthermore, turbidimetric measurements (Figure 7a) highlighted that in comparison to pristine HNTs [63], both nanomaterials present lower aqueous stability. To overcome these limitations, both HNTs/PB4 and HNTs-Calix/PB4 nanomaterials were used as fillers for Laponite^®^ (Lap) hydrogels. Lap, indeed, forms stable and thixotropic hydrogels in aqueous media due to the formation of delaminated dispersions by the self-assembling of its nanodisk via face-edge aggregation. Recently, it was demonstrated that the introduction of pristine, or covalently modified halloysite, as filler for Lap hydrogels led to the formation of stable composite hydrogels, which retain all properties of pristine ones. Similarly to previous studies [48], in the present case, the hydrogels retain the pristine Lap hydrogel properties. The hydrogels indeed demonstrated self-repair after disruption (Figure 7b,c), confirming their thixotropic nature, and they were stable to ultrasound irradiation. On the contrary, when PB4 was added directly to the Laponite^®^ dispersion without any supporting carrier, no stable hydrogel was formed (Figure 7d). These findings underline the essential role of HNTs and HNTs-Calix as mediators for drug incorporation into hydrogels. In addition to improving PB4 dispersion in aqueous environments, the nanocarriers act as physical bridges between the hydrophobic drug and the hydrophilic matrix, enabling stable network formation through interfacial interactions. Without such carriers, PB4 alone is unable to integrate into the gel structure due to its intrinsic incompatibility with the aqueous Laponite^®^ system.

Finally, to exploit the possibility to use the developed hydrogels as anticancer systems, their antiproliferative effects were evaluated on MCF-7 cancer cell lines by MTS assay. Both HNTs and Lap are not toxic in the concentration range investigated. The obtained IC_50_ values are reported in Table 3. As it is possible to observe, both hydrogels show promising cytotoxicity, with Lap/HNTs/PB_4_ system more active than Lap/HNTs-Calix/PB4 one. In comparison to free PB4 molecules, solubilized in DMSO, which showed an IC_50_ lower than 5 μM, the obtained results are very promising since they highlight the possibility to deliver PB4 in aqueous media and thus open up possible future use of it for in vivo tests.

The slightly higher cytotoxicity observed for Lap/HNTs/PB4 system compared to Lap/HNTs-Calix/PB4 may be attributed to differences in PB4 release kinetics. While calix[5]arene-functionalized HNTs ensure stronger host–guest interactions, which enhance PB4 encapsulation and stability, these interactions may also slow down the release of the drug under the assay conditions. Conversely, PB4 adsorbed on pristine HNTs is likely more readily available, leading to a more immediate cytotoxic response. These findings suggest that the two hydrogel systems provide complementary release profiles: Lap/HNTs/PB4 formulation ensures faster availability of the drug, while Lap/HNTs-Calix/PB4 system offers more controlled and sustained release. Such modulation capabilities could be strategically exploited depending on the therapeutic context, including potential combination therapies where timing and sequential drug delivery are critical.

From a translational perspective, halloysite-based nanocarriers offer several clinically relevant advantages, such as biocompatibility, scalability, and the possibility to be embedded in injectable hydrogels. The covalent functionalization with calixarene units enhances drug stabilization and release modulation, which are key for the therapeutic efficacy of hydrophobic and charged compounds. However, the clinical feasibility of the proposed system will depend on future investigations addressing biodistribution, in vivo safety, long-term stability, and regulatory aspects. These steps will be essential to evaluate the real potential of these hybrid platforms in localized anticancer therapies.

## 3. Materials and Methods

All reagents employed were purchased from Merck and used as received. Laponite^®^ XLG (Lap) was kindly donated by BYK (Wesel, Germany).

The NMR experiments were carried out at 27 °C using a Varian UNITY Inova 500 MHz spectrometer (^1^H at 499.88 MHz, ^13^C-NMR at 125.7 MHz) equipped with a pulse-field gradient module (Z axis) and a tunable 5 mm Varian inverse-detection probe (ID-PFG).

The HRMS experiments were performed using the LTQ XL mass spectrometer, equipped with H-ESI II source (ThermoFisher, San Jose, CA, USA), in full scan mode from *m*/*z* 150 to 2000. All measurements were performed in positive mode with a spray voltage of 3–3.5 kV. The capillary temperature was set to 250 °C, the capillary voltage to 20 V, and the tube lens to 120 V. External calibration was performed using the Pierce LTQ ESI Positive Ion Calibration Solution (ThermoFisher). Data proceeding was performed using the FreeStyle Software, ver. 1.6 SP1 (Thermo Fisher Scientific). All chemicals were reagent-grade and were used without further purification.

FT-IR spectra (KBr) were acquired by means of an Agilent Technologies Cary 630 FT-IR spectrometer (Santa Clara, CA, United States).

Thermogravimetric analyses were performed using a Perkin-Elmer TGA 7 instrument, equipped with a TAC 7/DX (Perkin Elmer, Waltham, MA, USA), applying a thermal ramp of 10 °C min^−1^ within the temperature range from 50 to 800 °C, under N_2_ atmosphere (60 mL min^−1^).

The size analysis, ζ-potential, and polydispersity index of the samples were determined using a Malvern Zetasizer Nano ZS instrument, fitted with a 632 nm laser at a fixed scattering angle of 173°.

Morphologies were imaged by means of a FEI Titan G2 60–300 ultra-high resolution transmission electron microscope (FEI, Lausanne, Switzerland) coupled with analytical electron microscopy (AEM) performed with an energy dispersive X-ray spectroscopy (XEDS) detector. AEM spectra were saved in scanning transmission electron microscopy (STEM) mode with a high angle annular dark field (HAADF) detector. Elemental maps were also collected using X-rays.

UV-*vis* measurements were acquired with a Beckmann DU 650 spectrometer.

Fluorescence measurements both in solution and in solid state were performed with a JASCO FP8300 spectrofluorometer. For measurements in solution, the excitation and emission slits were set at 5 nm and spectra were acquired in wavelength intervals ranging between 300 and 700 nm. Solid state spectra were acquired by setting the excitation and emission slits at 5 and 2.5 nm, respectively, in the wavelength interval ranging between 300 and 700 nm.

### 3.1. Synthesis of Propargyl-NH-Calix[5]

To a stirred solution of Calix[5]-NH_2_ (0.080 g, 0.067 mmol), dry CH_3_CN (20 mL) K_2_CO_3_ (0.037 g, 0.268 mmol), and propargyl tosylate (0.017 g, 0.080 mmol) were added. The solution was stirred at reflux, under nitrogen for 8 h. After checking the reaction by TLC (SiO_2_, Hexane/AcOEt 15:1, *v*/*v*, Rf = 0.70) to see the disappearance of the starting materials, the reaction mixture was allowed to cool to room temperature. The solid residue was dissolved in CH_2_Cl_2_, and the solution was washed with a solution of HCl 1 N (1 × 15 mL) and H_2_O (2 × 15 mL) and dried over Na_2_SO_4_. The solvent was evaporated under reduced pressure, and the residue was purified by column chromatography (SiO_2_, Hexane/AcOEt 40:1, *v*/*v*). The obtained product was triturated with CH_3_CN, producing the desired product, Propargyl-NH-Calix[5], as a yellowish powder (0.040 g, 0.033 mmol), with a yield of 48%.

^1^H-NMR (500 MHz, CDCl_3_) δ = 0.99, 1.25 (s, 1:1 ratio, *t*Bu, 36H), 0.93, 0.94 (d, *J* = 3.0 Hz, 1:2 ratio, CH(CH_3_)_2_, 30H), 1.21–1.38 (m, γ-CH_2_, 10H), 1.56–1.65 (m, δ-CH(CH_3_)_2_, 5H), 1.79–1.91 (m, β-CH_2_, 10H), 3.53–3.76 (m, α-CH_2_, 10H), 2.87 (bs, C=C-H, 1H) 3.17 (s, CH_2_-C=C, 2H), 3.19 and 4.47 (AX, *J* = 14 Hz, ArCH_2_Ar, 4H), 3.24 and 4.53 (AX, *J* = 13 Hz, ArCH_2_Ar, 2H), 3.26 and 4.56 (AX, *J* = 14 Hz, ArCH_2_Ar, 2H), 5.29 (s, C5-NH-Prop, 1H), 5.77 (s, ArH-C5, 2H), 6.82 and 6.83 (ABq, *J* = 3 Hz, ArH, 4 H), 7.06 and 7.16 (ABq, *J* = 3 Hz, ArH, 4 H) ppm.

^13^C-NMR (125 MHz, CDCl_3_): δ = 22.6, 22.7, 22.8 (×2), 22.85 CH(**C**H_3_)_2_], 28.13, 28.16, 28.2 (γ-CH_2_), 28.3, 28.4, 28.6 [**C**H(CH_3_)_2_], 28.7, 30.0 (Ar**C**H_2_Ar), 31.2, 31.6, 33.3, 33.9 (*t*Bu), 34.0, 35.0, 35.2 (β-CH_2_), 35.3 (NH-CH_2_), 70.9 (C alkyne), 74.0, 74.3, 74.5 (α-CH_2_), 112.7 (CH alkyne), 125.0, 125.3, 125.5, 125.9, 126.4 (*o*-Ar), 133.5, 133.7, 133.9, 134.1, 134.3 (*m*-Ar), 141.4, 144.5, 144.6 (*p*-Ar), 147.5, 152.8, 153.4 (*ipso*-Ar). ESI-MS 1228 *m*/*z* = [M]^+^; 1246 *m*/*z* = [M + NH_4_]^+^; 1251 *m*/*z* = [M + Na]^+^; 1267 *m*/*z* = [M + K]^+^.

### 3.2. Synthesis of HNTs-Calix Nanomaterial

In a 10 mL round bottom flask, 100 mg of HNTs-N_3_, 15 mg of Propargyl-NH-Calix[5] (0.012 mmol), and 4 mL of a mixture of H_2_O/^t^BuOH (1:1) were added. To the obtained dispersion, 0.100 mL of a CuSO_4_ aqueous solution (1 M) and 1.0 mL of an aqueous solution of sodium ascorbate (1 M) were added. The mixture was left to stir at room temperature for ca. 24 h. Then the solid was washed several times with methanol and dried at 60 °C overnight.

### 3.3. Loading of PB4 into HNTs or HNTs-Calix Nanomaterials

To a dispersion of HNTs or HNTs-Calix nanomaterials in H_2_O (15 mL), 3 mL of a 3.2 mM solution of PB4 in ACN was added. The obtained suspension was sonicated for 5 min and then evacuated for 3 cycles. The suspension was left under stirring for 16 h at room temperature. After this time, the powder was washed with water until unreacted PB4 was removed and then dried under vacuum at 60 °C.

### 3.4. Kinetic Release of PB4 from HNTs/PB4 and HNTs-Calix/PB4 Nanomaterials

For the release of PB4 from the HNTs and HNTs-Calix nanomaterial, 10 mg of the corresponding sample was dispersed in 1 mL of release medium (HCl 0.1 M, pH 1.0 or H_2_O, pH 7.0) and transferred into a sealed dialysis membrane (Medicell Membrane International Ltd. (Greenwich, London, UK) MWCO 12–14,000 Da with a diameter of 21.5 mm). Afterwards, the membrane was put in a round bottom flask containing 9 mL of the release medium, maintained at 37 °C under constant stirring. At a fixed time, 1 mL of the medium was withdrawn and analyzed by UV-*vis* measurement. To ensure sink conditions, 1 mL of fresh solution was used to replace the collected one. The total amount of PB4 released (*F_t_*) was calculated as follows:

(1) $$F_{t}=V_{m}C_{t}+\sum_{i=0}^{t-1}V_{a}C_{i}$$

where *V_m_* and *C_t_* are the volume and the concentration of the molecule at time *t*. *V_a_* is the volume of the sample withdrawn and *C_i_* is the PB4 concentration at time *i* (*i* < *t*). The kinetic release was analyzed by the following models:

(2) $$Power\;fit\text{:}\;F_{t}={kt}^{n}$$

(3) $$First\text{-}order\text{:}\;F_{t}=M_{\infty }\cdot \left({1-e}^{-kt}\right)$$

(4) $$DEM\text{:}\;F_{t}=M^{\prime }\cdot \left({1-e}^{-k^{\prime }t}\right)+M^{\prime \prime }\left({1-e}^{-k\prime \prime t}\right)$$

### 3.5. Adsorption Isotherm

The adsorption isotherm of HNTs was obtained by weighing 3 mg of HNTs in the presence of PB4 solution in a concentration range of 5 × 10^−6^–1 × 10^−4^ M, which was then shaken in a thermostated shaker at 200 rpm for 12 h to reach equilibrium at 298 K. The equilibrium adsorption capacity *Q_e_* (mol g^−1^) was calculated by the following equation:

(5) $$Q_{e}=\frac{(C_{0}-C_{e})\cdot V}{M}$$

where *C*_0_ and *C_e_* are initial end equilibrium concentrations of PB4 (M), respectively, *M* is the weight of HNTs (g), and *V* is the volume of PB4 solution (L).

### 3.6. Lap/HNTs/PB4 and Lap/HNTs-Calix/PB4 Hydrogel Preparation

The hydrogels were prepared by weighing 3.7 mg of PB4, HNTs/PB4, or HNTs-Calix/PB4 and 980 µL of H_2_O into screw-capped vials (diameter 1.0 cm). The mixture was dispersed for 2 min using ultrasound and left to stir at room temperature (1000 rpm for 5 min). After this time, 20 mg of Lap XLG was added to each vial under constant stirring, and the vials were left to stir at room temperature for 24 h.

### 3.7. Thixotropic and Sonotropic Behavior

All the hydrogels obtained were subjected to a mechanical stimulus using a stirring bar of 6 mm in length and 2 mm in height (1000 rpm for 5 min). Additionally, the hydrogels were placed in an ultrasound water bath for 5 min, with a power of 200 W and a frequency of 45 kHz. After 30 min at room temperature, the gels were observed to evaluate their thixotropic properties, and following the tube-inversion test, they were classified as thixotropic.

### 3.8. Cell Lines

Breast adenocarcinoma cells MCF-7 were acquired from ATCC (HTB-22, Rockville, MD, USA) and cultured in DMEM (Dulbecco’s modified Eagle’s medium; HyClone Europe Ltd., Cramlington, UK). The medium was completed with 10% heat-inactivated fetal calf serum, 2 mM l-glutamine, 100 U/mL penicillin, and 100 μg/mL streptomycin (all reagents were from HyCloneEurope). MCF-7 cells were cultured in a humidified atmosphere of 5% CO_2_ at 37 °C and used after trypsinization in a narrow range of passage numbers for the experiments.

### 3.9. Cell Viability Assay

MCF-7 cells are seeded at 5 × 10^3^ cells/well in 96-well plates and incubated overnight at 37 °C. The medium was replaced with fresh complete medium supplemented with different compounds at several concentrations. After 72 h of treatment, MTS dye was added (16 µL). Then, 96-well plates were incubated in an incubator at 37 °C in 5% CO_2_ for 2 h, and the bioreduction of the MTS reagent was obtained by measuring the absorbance at 490 nm through a microplate reader (iMark Microplate Reader; Bio-Rad Laboratories Inc., Hercules, CA, USA).

## 4. Conclusions

In this work, we developed and characterized two halloysite-based hybrid nanomaterials designed for the encapsulation and delivery of hydrophobic, positively charged anticancer agents. To the best of our knowledge, this is the first report of the covalent functionalization of halloysite nanotubes with calix[5]arene units, creating a novel multicavity architecture that combines lumen encapsulation with external supramolecular recognition. PB4, a poorly water-soluble planar aromatic compound with potent antiproliferative activity, was used as a model system to evaluate the loading efficiency, aqueous stability, and cytotoxic behavior of the new materials. Both HNTs/PB4 and HNTs-Calix/PB4 were successfully incorporated into thixotropic Laponite^®^-based hydrogels, which preserved their mechanical properties and injectability. The cytotoxic assays demonstrated that both hydrogel formulations were active against MCF-7 breast cancer cells, with the two systems displaying different release behaviors. The possibility to modulate drug release profiles through tailored surface functionalization opens the way to the rational design of adaptable nanocarriers for monotherapy or combination therapies. These results lay the groundwork for further in vivo studies on injectable clay-based platforms for the delivery of poorly soluble anticancer drugs. However, future investigations are needed to evaluate the long-term stability and in vivo performance of the thixotropic hydrogels developed to assess their potential clinical feasibility.

## Figures and Tables

**Table 1:** Parameters of Langmuir and Freundlich Isotherm models for PB4 adsorption on HNTs.

Langmuir			Freundlich		
Q_m_ (10^−5^ mol g^−1^)	K_L_ (10^5^ L mol^−1^)	R^2^	K_F_ (10^−4^ mol g^−1^ (mol L^−1^)^1/*n*^)	*n*	R^2^
7 ± 1	1.4 ± 0.9	0.8623	9 ± 3	3.7 ± 0.4	0.9825

**Table 2:** Kinetic parameters for PB4 release from HNTs/PB4 and HNTs-Calix/PB4 at pH 1.0 and pH 7.4.

Nanomaterial	pH	DEM			First Order		Power Fit		
		*k*_1_ (min^−1^)	*k*_2_ (min^−1^)	R^2^	*k*_1_ (min^−1^)	R^2^	*k*_F_ (min^−1^)	*n*	R^2^
HNTs/PB4	1.0	n.a.	n.a.	/	0.026 ± 0.002	0.9806	12 ± 1	0.42 ± 0.03	0.9746
	7.0	n.a.	n.a.	/	0.0038 ± 0.0005	0.9473	0.26 ± 0.03	0.43 ± 0.02	0.9799
HNTs-Calix/PB4	1.0	0.038 ± 0.001	0.0017 ± 0.0006	0.9974	0.029 ± 0.002	0.9796	8 ± 1	0.02 ± 0.03	0.8765
	7.0	0.06 ± 0.01	0.0017 ± 0.0004	0.9888	0.013 ± 0.003	0.8792	0.38 ± 0.03	0.30 ± 0.01	0.9894

**Table 3:** IC_50_ values of developed Lap hydrogels.

Nanomaterials	IC_50_ (µM)
Lap/HNTs/PB4	10.0
Lap/HNTs-Calix/PB4	21.0

## Data Availability

Dataset available on request from the authors.

## Supplementary Materials

The following supporting information can be downloaded at: https://www.mdpi.com/article/10.3390/ph18091356/s1, Figure S1: synthesis of PB4 molecule, Figure S2: TEM image of pristine HNT and HNT diameter size distribution, Figure S3: Synthesis of Propargyl-NH-Calix[5]. References [39,56,64] have been cited in the Supplementary Materials.

## References

[1] Kumar P., Mangla B., Javed S., Ahsan W., Musyuni P., Sivadasan D., Alqahtani S.S., Aggarwal G. (2023). A review of nanomaterials from synthetic and natural molecules for prospective breast cancer nanotherapy. Front. Pharmacol..

[2] Nicosia A., Vento F., Satriano C., Villari V., Micali N., Cucci L.M., Sanfilippo V., Mineo P.G. (2020). Light-Triggered Polymeric Nanobombs for Targeted Cell Death. ACS Appl. Nano Mater..

[3] Massaro M., Borrego-Sánchez A., Viseras-Iborra C., Cinà G., García-Villén F., Liotta L.F., Lopez Galindo A., Pimentel C., Sainz-Díaz C.I., Sánchez-Espejo R. (2024). Hectorite/Phenanthroline-Based Nanomaterial as Fluorescent Sensor for Zn Ion Detection: A Theoretical and Experimental Study. Nanomaterials.

[4] Zhuo Y., Zhao Y.G., Zhang Y. (2024). Enhancing Drug Solubility, Bioavailability, and Targeted Therapeutic Applications through Magnetic Nanoparticles. Molecules.

[5] Li K., Guo B., Gu J., Ta N., Gu J., Yu H., Sun M., Han T. (2025). Emerging advances in drug delivery systems (DDSs) for optimizing cancer complications. Mater. Today Bio.

[6] Gupta P., Neupane Y.R., Parvez S., Kohli K. (2021). Recent Advances in Targeted Nanotherapeutic Approaches for Breast Cancer Management. Nanomedicine.

[7] Nicosia A., La Perna G., Cucci L.M., Satriano C., Mineo P. (2022). A Multifunctional Conjugated Polymer Developed as an Efficient System for Differentiation of SH-SY5Y Tumour Cells. Polymers.

[8] Vento F., Privitera A., Caruso G., Nicosia A. (2025). A Silibinin-Poly(ε-Caprolactone) Conjugate as an Enhanced Anticancer Agent. Macromol. Biosci..

[9] You W., Cai Z., Xiao F., Zhao J., Wang G., Wang W., Chen Z., Hu W., Chen Y., Wang Z. (2025). Biomolecular Microneedle Initiates Fe_3_O_4_/MXene Heterojunction-Mediated Nanozyme-Like Reactions and Bacterial Ferroptosis to Repair Diabetic Wounds. Adv. Sci..

[10] Padil V.V.T., Akshay Kumar K.P., Murugesan S., Torres-Mendieta R., Wacławek S., Cheong J.Y., Černík M., Varma R.S. (2022). Sustainable and safer nanoclay composites for multifaceted applications. Green Chem..

[11] Peixoto D., Pereira I., Pereira-Silva M., Veiga F., Hamblin M.R., Lvov Y., Liu M., Paiva-Santos A.C. (2021). Emerging role of nanoclays in cancer research, diagnosis, and therapy. Coord. Chem. Rev..

[12] Stavitskaya A., Khusnetdenova E., Vinokurov V., Lvov Y., Fakhrullin R. (2022). Prokaryotic and eukaryotic toxicity of halloysite decorated with photoactive nanoparticles. Chem. Commun..

[13] Rozhina E., Panchal A., Akhatova F., Lvov Y., Fakhrullin R. (2020). Cytocompatibility and cellular uptake of alkylsilane-modified hydrophobic halloysite nanotubes. Appl. Clay Sci..

[14] Cardano F., Massaro M., Leone F., Cinà G., Borbone N., Falanga A.P., Oliviero G., Nicosia A., Fresia M., Fin A. (2025). Halloysite Nanotubes Functionalized with Naphthalene Diimide and Peptide Nucleic Acid Derivatives: Toward Multifunctional Nanomaterials. ACS Appl. Nano Mater..

[15] Feng Y., Chen X., He R.-R., Liu Z., Lvov Y.M., Liu M. (2024). The Horizons of Medical Mineralogy: Structure-Bioactivity Relationship and Biomedical Applications of Halloysite Nanoclay. ACS Nano.

[16] Caruso M.R., Calvino M.M., Cavallaro G., Amato J., Marzano S., D’Aria F., Giancola C., Lazzara G., Milioto S., Pagano B. (2025). Halloysite clay nanotubes as platforms for loading of aptamers and antisense oligonucleotides. Hybrid Adv..

[17] Chen X., Feng Y., Zhang D., Zhou S., Liu X., Luo B., Zhou C., Liu M. (2025). Orally administered hydrogel containing polyphenol@halloysite clay for probiotic delivery and treatment of inflammatory bowel disease. Nano Today.

[18] Drits V.A., Sakharov B.A., Hillier S. (2018). Phase and structural features of tubular halloysite (7 Å). Clay Miner..

[19] Pasbakhsh P., Churchman G.J., Keeling J.L. (2013). Characterisation of properties of various halloysites relevant to their use as nanotubes and microfibre fillers. Appl. Clay Sci..

[20] Wilson I., Keeling J. (2016). Global occurrence, geology and characteristics of tubular halloysite deposits. Clay Miner..

[21] Glotov A., Vutolkina A., Pimerzin A., Vinokurov V., Lvov Y. (2021). Clay nanotube-metal core/shell catalysts for hydroprocesses. Chem. Soc. Rev..

[22] Cinà G., Massaro M., Cavallaro G., Lazzara G., Sánchez-Espejo R., Viseras Iborra C., D’Abrosca B., Fiorentino A., Messina G.M.L., Riela S. (2024). Development of alginate film filled with halloysite-carbon dots for active food packaging. Int. J. Biol. Macromol..

[23] Viscusi G., Boccalon E., Lamberti E., Nocchetti M., Gorrasi G. (2024). Alginate Microbeads Containing Halloysite and Layered Double Hydroxide as Efficient Carriers of Natural Antimicrobials. Nanomaterials.

[24] Jiang Z., Sun S., Liu J., Sun X. (2024). Recent Advances of Halloysite Nanotubes in Biomedical Applications. Small.

[25] Fizir M., Dramou P., Dahiru N.S., Ruya W., Huang T., He H. (2018). Halloysite nanotubes in analytical sciences and in drug delivery: A review. Microchim. Acta.

[26] Farokh A., Pourmadadi M., Rashedi H., Yazdian F., Navaei-Nigjeh M. (2023). Assessment of synthesized chitosan/halloysite nanocarrier modified by carbon nanotube for pH-sensitive delivery of curcumin to cancerous media. Int. J. Biol. Macromol..

[27] Massaro M., Cinà G., Cavallaro G., Lazzara G., Silvestri A., Barbosa R.d.M., Sànchez-Espejo R., Viseras-Iborra C., Notarbartolo M., Riela S. (2024). Comparison of Synthetic Pathways for Obtaining Fluorescent Nanomaterials Based on Halloysite and Carbon Dots for Potential Biological Sensing. Int. J. Mol. Sci..

[28] Hamedinasab H., Sabahi H., Hosseini M., Rezayan A.H. (2025). Formulation, optimization, and characterization of naringenin-loaded halloysite nanotube to achieve enhanced antioxidant and anticancer properties. Nanomed. J..

[29] Boraei S.B.A., Eshghabadi F., Hosseinpour R., Zare Y., Munir M.T., Rhee K.Y. (2024). Halloysite nanotubes in biomedical applications: Recent approaches and future trends. Appl. Clay Sci..

[30] Massaro M., Ciani R., Grossi G., Cavallaro G., de Melo Barbosa R., Falesiedi M., Fortuna C.G., Carbone A., Schenone S., Sánchez-Espejo R. (2024). Halloysite Nanotube-Based Delivery of Pyrazolo[3,4-d]pyrimidine Derivatives for Prostate and Bladder Cancer Treatment. Pharmaceutics.

[31] Li X., Chen J., Liu H., Deng Z., Li J., Ren T., Huang L., Chen W., Yang Y., Zhong S. (2019). β-Cyclodextrin coated and folic acid conjugated magnetic halloysite nanotubes for targeting and isolating of cancer cells. Colloids Surf. B Biointerfaces.

[32] Massaro M., Riela S. (2018). Organo-Clay Nanomaterials Based on Halloysite and Cyclodextrin as Carriers for Polyphenolic Compounds. J. Funct. Biomater..

[33] Español E.S., Villamil M.M. (2019). Calixarenes: Generalities and Their Role in Improving the Solubility, Biocompatibility, Stability, Bioavailability, Detection, and Transport of Biomolecules. Biomolecules.

[34] Farber M., Rawat V., Diskin-Posner Y., Dobrovetsky R., Vigalok A. (2024). Polyaromatic Calixarene Hosts: Calix[4]pyrenes. Org. Lett..

[35] Lhoták P. (2024). Upper rim-bridged calixarenes. RSC Adv..

[36] Dai Y., Yu W., Cheng Y., Zhou Y., Zou J., Meng Y., Chen F., Qian Y., Yao Y. (2025). Recent developments in pillar[5]arene-based nanomaterials for cancer therapy. Chem. Commun..

[37] Álvarez-Yebra R., López-Coll R., Clos-Garrido N., Lozano D., Lledó A. (2024). Calix[5]arene Self-Folding Cavitands: A New Family of Bio-Inspired Receptors with Enhanced Induced Fit Behavior. Isr. J. Chem..

[38] Carpentier R., Testa C., Pappalardo A., Jabin I., Bartik K. (2025). Binding of Bioactive Ammonium Ions in Water with a Cavity-Based Selectivity: Water Solubilization versus Micellar Incorporation. J. Org. Chem..

[39] Testa C., Gangemi C.M.A., Sfrazzetto G.T., Ricceri M., Giuffrida A., Greco V., Cancelliere A.M., Puntoriero F., Pappalardo A. (2024). Luminescent Dansyl-Calix[5]arene for the Recognition of Biogenic Amines. Curr. Org. Chem..

[40] Minh Hoang C.N., Nguyen S.H., Tran M.T. (2025). Nanoparticles in cancer therapy: Strategies to penetrate and modulate the tumor microenvironment—A review. Smart Mater. Med..

[41] Gajbhiye K.R., Salve R., Narwade M., Sheikh A., Kesharwani P., Gajbhiye V. (2023). Lipid polymer hybrid nanoparticles: A custom-tailored next-generation approach for cancer therapeutics. Mol. Cancer.

[42] Prieložná J., Mikušová V., Mikuš P. (2024). Advances in the delivery of anticancer drugs by nanoparticles and chitosan-based nanoparticles. Int. J. Pharm. X.

[43] Hani U., Choudhary V.T., Ghazwani M., Alghazwani Y., Osmani R.A.M., Kulkarni G.S., Shivakumar H.G., Wani S.U.D., Paranthaman S. (2024). Nanocarriers for Delivery of Anticancer Drugs: Current Developments, Challenges, and Perspectives. Pharmaceutics.

[44] Barresi V., Bonaccorso C., Consiglio G., Goracci L., Musso N., Musumarra G., Satriano C., Fortuna C.G. (2013). Modeling, design and synthesis of new heteroaryl ethylenes active against the MCF-7 breast cancer cell-line. Mol. Biosyst..

[45] Bongiorno D., Musso N., Bonacci P.G., Bivona D.A., Massimino M., Stracquadanio S., Bonaccorso C., Fortuna C.G., Stefani S. (2021). Heteroaryl-Ethylenes as New Lead Compounds in the Fight against High Priority Bacterial Strains. Antibiotics.

[46] Brunchi C.-E., Morariu S. (2024). Laponite^®^—From Dispersion to Gel—Structure, Properties, and Applications. Molecules.

[47] Hu Y., Wang Y., Liu Y., Dai J., Wang J., Ju C. (2025). Injectable laponite nanocomposite hydrogel with synergistic antibacterial and odontogenic activity for endodontic regeneration. Colloids Surf. B Biointerfaces.

[48] Massaro M., Cinà G., Borrego-Sánchez A., Sainz-Díaz C.I., Viseras-Iborra C., Sánchez-Espejo R., de Melo Barbosa R., Leone F., Pibiri I., Noto R. (2024). Thixotropic Hydrogels Based on Laponite^®^ and Cucurbituril for Delivery of Lipophilic Drug Molecules. ChemPlusChem.

[49] Stealey S.T., Gaharwar A.K., Zustiak S.P. (2023). Laponite-Based Nanocomposite Hydrogels for Drug Delivery Applications. Pharmaceuticals.

[50] Vigdorowitsch M., Pchelintsev A., Tsygankova L., Tanygina E. (2021). Freundlich Isotherm: An Adsorption Model Complete Framework. Appl. Sci..

[51] Gereli G., Seki Y., Murat Kuşoğlu İ., Yurdakoç K. (2006). Equilibrium and kinetics for the sorption of promethazine hydrochloride onto K10 montmorillonite. J. Colloid Interface Sci..

[52] Duce C., Vecchio Ciprioti S., Ghezzi L., Ierardi V., Tinè M.R. (2015). Thermal behavior study of pristine and modified halloysite nanotubes. J. Therm. Anal. Calorim..

[53] Lisuzzo L., Cavallaro G., Milioto S., Lazzara G. (2022). Halloysite nanotubes as nanoreactors for heterogeneous micellar catalysis. J. Colloid Interface Sci..

[54] Yuan P., Southon P.D., Liu Z., Green M.E.R., Hook J.M., Antill S.J., Kepert C.J. (2008). Functionalization of Halloysite Clay Nanotubes by Grafting with γ-Aminopropyltriethoxysilane. J. Phys. Chem. C.

[55] Stetefeld J., McKenna S.A., Patel T.R. (2016). Dynamic light scattering: A practical guide and applications in biomedical sciences. Biophys. Rev..

[56] Garozzo D., Gattuso G., Notti A., Pappalardo A., Pappalardo S., Parisi M.F., Perez M., Pisagatti I. (2005). A Calix[5]arene-Based Heterotetratopic Host for Molecular Recognition of Long-Chain, Ion-Paired α,ω-Alkanediyldiammonium Salts. Angew. Chem. Int. Ed..

[57] Görner H., Gruen H. (1985). Photophysical properties of quaternary salts of 4-dialkylamino-4′-azastilbenes and their quinolinium analogues in solution: IX. J. Photochem..

[58] Fromherz P., Heilemann A. (1992). Twisted internal charge transfer in (aminophenyl)pyridinium. J. Phys. Chem..

[59] Carlotti B., Benassi E., Spalletti A., Fortuna C.G., Elisei F., Barone V. (2014). Photoinduced symmetry-breaking intramolecular charge transfer in a quadrupolar pyridinium derivative. Phys. Chem. Chem. Phys..

[60] Benassi E., Carlotti B., Fortuna C.G., Barone V., Elisei F., Spalletti A. (2015). Acid–Base Strength and Acidochromism of Some Dimethylamino–Azinium Iodides. An Integrated Experimental and Theoretical Study. J. Phys. Chem. A.

[61] Bretti C., Cataldo S., Gianguzza A., Lando G., Lazzara G., Pettignano A., Sammartano S. (2016). Thermodynamics of Proton Binding of Halloysite Nanotubes. J. Phys. Chem. C.

[62] Espíndola C., Correa A.J., López-López M., López-Cornejo P., Bernal E., Lebrón J.A., Ostos F.J., Benhnia M.R.-E.-I., Moyá M.L. (2022). Single -and Multi-Walled Carbon Nanotubes as Nanocarriers for the Delivery of 7-Hydroxyflavone. Pharmaceutics.

[63] Mancuso A., Massaro M., Leone F., Bonaccorsi P.M., Compagnini G., Gangemi C.M.A., Puntoriero F., Ribagorda M., Scardaci V., Viseras C. (2025). Glucosyl OPE-Modified Halloysite Nanotubes and Their Potential as Phototherapy Agents for Bacterial Infections. Surf. Interfaces.

[64] Fortuna C.G., Barresi V., Bonaccorso C., Consiglio G., Failla S., Trovato-Salinaro A., Musumarra G. (2012). Design, synthesis and in vitro antitumour activity of new heteroaryl ethylenes. Eur. J. Med. Chem..

