# Supplementary material for: Covalently Functionalized Halloysite-Calixarene Nanotubes for Injectable Hydrogels: A Multicavity Platform for Hydrophobic Drug Delivery
Source: Pharmaceuticals (Basel). 2025 Sep 11;18(9):1356. doi: 10.3390/ph18091356 (PMC12472301; doi:10.3390/ph18091356)
Supplement: Supplementary file 1 [file pharmaceuticals-18-01356-s001.zip › pharmaceuticals-3803881-supplementary.pdf]

Supporting Information

Covalently Functionalized Halloysite-Calixarene Nanotubes for Injectable Hydrogels: A Multicavity Platform for Hydrophobic Drug Delivery

Giuseppe Cinà[a], Marina Massaro[a], Andrea Pappalardo[b,c,*], Carmela Bonaccorso[b], Cosimo G. Fortuna[b], Placido G. Mineo[b,d], Angelo Nicosia[b], Paola Poma[a], Rita Sánchez-Espejo[e], Caterina Testa[b], César Viseras[e,f], and Serena Riela[b,*]

[a] Dipartimento di Scienze e Tecnologie Biologiche, Chimiche e Farmaceutiche (STEBICEF), Università di Palermo, Viale delle Scienze, Parco d'Orleans II, Ed. 17, 90128 Palermo, Italy.

[b] Dipartimento di Scienze Chimiche, Università di Catania, Catania 95125, Italy. E-mails: andrea.pappalardo@unict.it; serena.riela@unict.it

[c] INSTM, UdR di Catania, Catania 95125, Italy.

[d] Istituto per i Processi Chimico-Fisici, Consiglio Nazionale delle Ricerche (IPCF-CNR), Viale F. Stagno d'Alcontres 37, I-98158, Messina, Italia.

[e] Department of Pharmacy and Pharmaceutical Technology, Faculty of Pharmacy, University of Granada, Campus Universitario de Cartuja, 18071 Granada, Spain

[f] Andalusian Institute of Earth Sciences, CSIC-UGR, 18100 Armilla, Granada, Spain

Total pages numbers: 6

Total Figures: 3

Table of content

1. Synthesis of PB4 molecule……………………………………………………………...S3
2. TEM image of pristine HNT; (b) HNT diameter size distribution………………………...S4
3. Synthesis of Propargyl-NH-Calix[5]………………………………………………………S5
4. References…………………………………………………………………………………S6

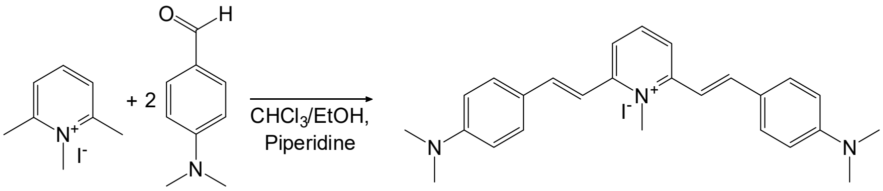

Figure S1. Synthesis of PB4 [3].

(a)
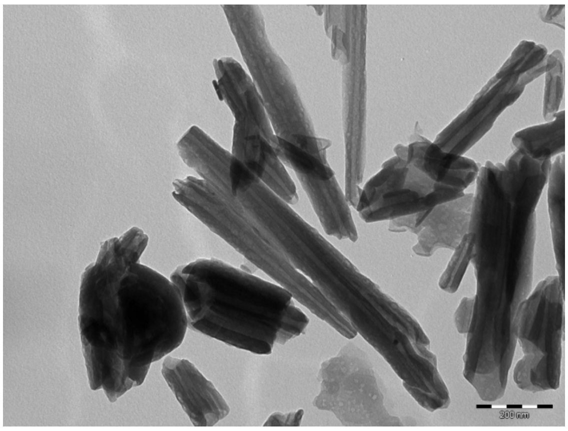

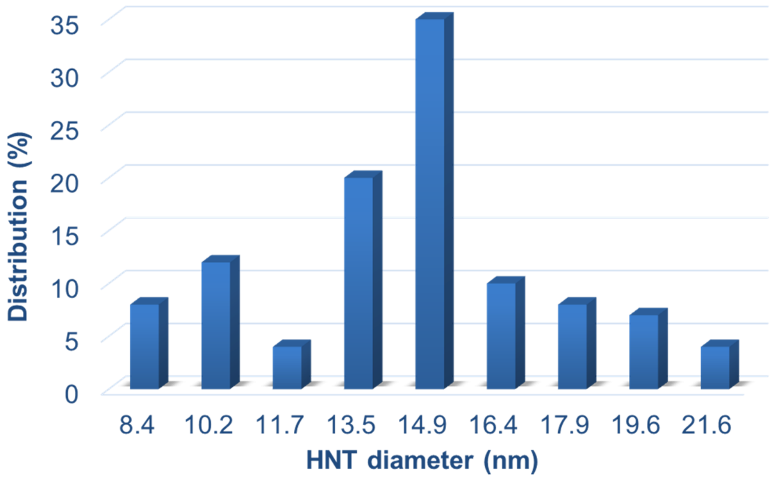

(b)

Figure S2. (a) TEM image of pristine HNT; (b) HNT diameter size distribution (n=40).

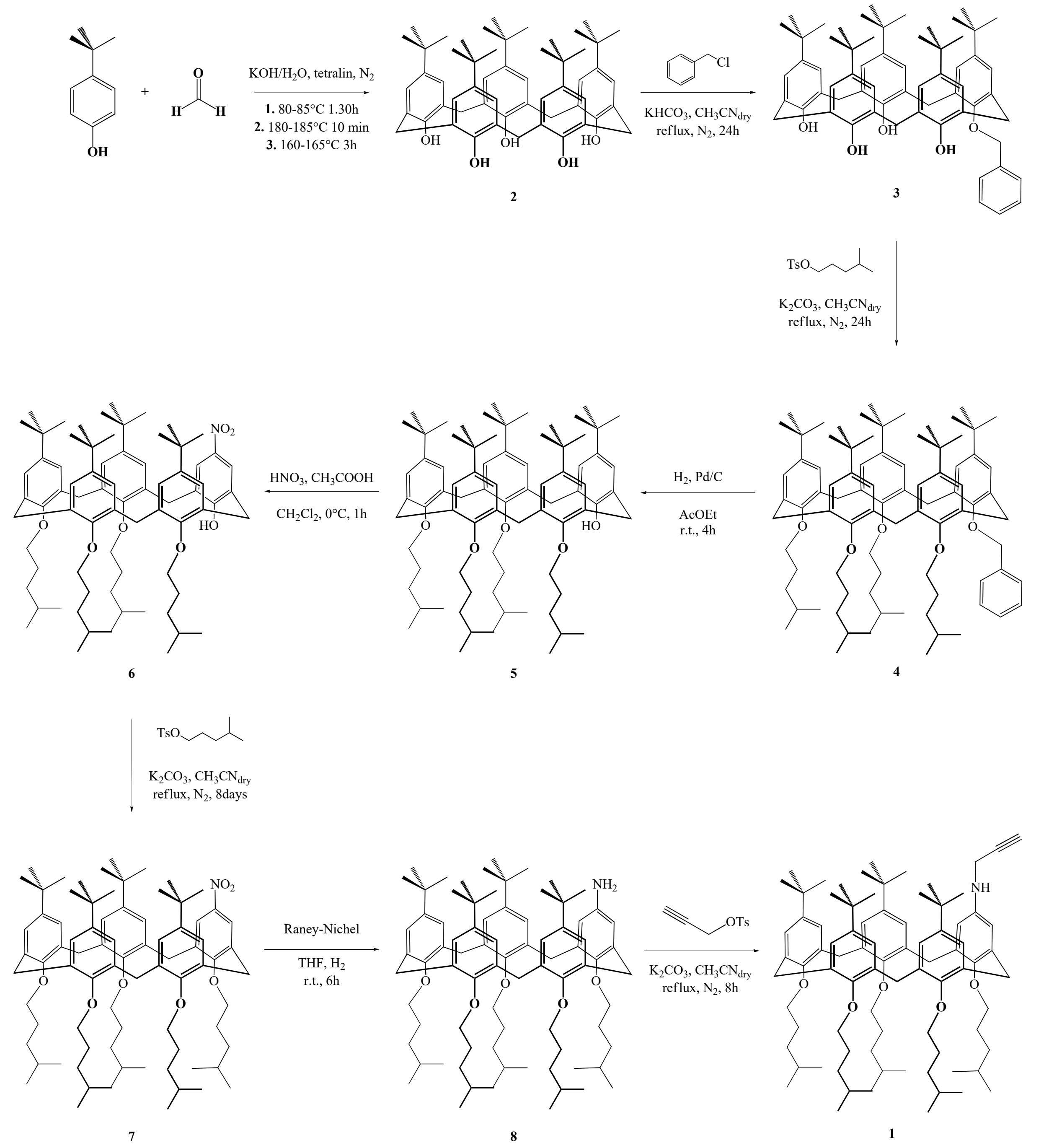

Figure S3. Synthesis of NH2-Calix[5] [1, 2].

References

[1] D. Garozzo, G. Gattuso, A. Notti, A. Pappalardo, S. Pappalardo, M.F. Parisi, M. Perez, I. Pisagatti, Angew. Chemie Int. Ed. 44 (2005) 4892-4896.

[2] C. Testa, C.M.A. Gangemi, G.T. Sfrazzetto, M. Ricceri, A. Giuffrida, V. Greco, A.M. Cancelliere, F. Puntoriero, A. Pappalardo, Curr. Org. Chem., 28 (2024) 1380-1386.

[3] C. G. Fortuna, V. Barresi, C. Bonaccorso, G. Consiglio, S. Failla, A. Trovato-Salinaro, G. Musumarra, Eur. J. Med. Chem. 47 (2022) 221-227. DOI: 10.1016/j.ejmech.2011.10.060.
